# Stimulants associated with reduced risk of hospitalization for motor vehicle accident injury in patients with obstructive sleep apnea-a nationwide cohort study

**DOI:** 10.1186/s12890-019-1041-1

**Published:** 2020-02-03

**Authors:** Yi-Chang Lin, Tien-Yu Chen, Wu-Chien Chien, Chi-Hsiang Chung, Hsin-An Chang, Yu-Chen Kao, Chien-Sung Tsai, Chih-Sheng Lin, Nian-Shen Tzeng

**Affiliations:** 1 Division of Cardiovascular Surgery, Department of Surgery, Tri-Service General Hospital, National Defense Medical Center, Taipei, Taiwan; 2 Department of Biological Science and Technology, National Chiao Tung University, No.75, Po-Ai Street, Hsinchu, 30068 Taiwan; 3 Department of Psychiatry, Tri-Service General Hospital, School of Medicine, National Defense Medical Center, No. 325, Section 2, Cheng-Kung Road, Neihu District, Taipei City, 11490 Taiwan; 4 Department of Psychiatry, Tri-Service General Hospital, Keelung Branch, National Defense Medical Center, Taipei, Taiwan; 5 Department of Medical Research, Tri-Service General Hospital, National Defense Medical Center, Taipei, Taiwan; 6 School of Public Health, National Defense Medical Center, Taipei, Taiwan; 7 Graduate Institute of Life Sciences, National Defense Medical Center, Taipei, Taiwan; 8 Taiwanese Injury Prevention and Safety Promotion Association, Taipei, Taiwan; 9 Student Counseling Center, National Defense Medical Center, Taipei, Taiwan; 10 Department of Psychiatry, Tri-Service General Hospital, Song-Shan Branch, National Defense Medical Center, Taipei, Taiwan

**Keywords:** Obstructive sleep apnea, Motor vehicle accident injury, Cohort study, National Health Insurance Research Database, Longitudinal health insurance database

## Abstract

**Background:**

The risk of injury directly related to hospitalization for motor vehicle accidents (MVAs) in the obstructive sleep apnea (OSA) patients has not been thoroughly understood. Our study aimed to examine the association between the OSA and the hospitalization for an MVA injury.

**Methods:**

This retrospective cohort study used Taiwan’s National Health Insurance Research Database (NHIRD) between 2000 and 2015. The OSA patients aged ≥20 years by age, sex, and index-year matched by non-OSA controls were enrolled (1:3). We used the Cox proportional regression model to evaluate the association between the OSA and the hospitalization for an MVA injury.

**Results:**

The incidence rate of hospitalization for an MVA injury was higher in the OSA cohort (*N* = 3025) when compared with the non-OSA controls (*N* = 9075), as 575.3 and 372.0 per 100,000 person-years, respectively (*p* < 0.001). The Kaplan-Meier analysis showed that the OSA cohort had a significantly higher incidence of hospitalization for the MVA injury (log-rank test, *p* < 0.001). After adjusting for the covariates, the risk of hospitalization for the MVA injury among the OSA was significantly higher (hazard ratio [HR] =2.18; 95% confidence interval [CI] = 1.79–2.64; *p* < 0.001). Stimulants usage was associated with a nearly 20% decrease in the risk of an overall hospitalization for an MVA injury in the OSA patients.

**Conclusions:**

This study provides evidence that patients with OSA are at a two-fold higher risk of developing hospitalization for an MVA injury, and the usage of modafinil and methylphenidate was associated with a lower risk of an overall hospitalization for the MVA injury.

## Background

Obstructive sleep apnea (OSA) is a common disorder characterized by repeated partial, or the complete collapse of, the upper airway with episodes of apnea and hypopnea during sleep [1]. OSA affects 3–7% adults in general adults and up to 9–15% of middle-aged adults. According to the varying criteria of diagnostic studies, with a male predominance [2, 3]. Excessive daytime sleepiness (EDS) and cognitive performance from OSA are associated with occupational injury and hospitalization for motor vehicle accidents (MVAs) [4].

An MVA injury is a major global public health problem, which has accounted for mortality, morbidity and economic loss [5, 6]. Sleep disorders have been related to MVAs in previous researches [7, 8]. One study also found that problems such as sleepiness would increase the risk of near-miss accidents [9], and another study showed that drivers with sleep-related near misses may predict MVAs [8]. The OSA patients have a higher risk of falling asleep while driving and are 3-fold more likely to cause MVA injuries [10, 11], thus, hypersomnolence related to OSA was associated with a high risk when driving [7]. Previous studies have also shown that treating OSA, for example, with continuous positive airway pressure (CPAP) could reduce the risk of an MVA, costs, and fatalities related to these MVAs [10, 12].

One cross-sectional study has found that OSA, sleep debt, and EDS are independently associated with road accidents in truck drivers [13]. Another study has shown that about 7% of road traffic accident injuries for the male drivers involved in MVAs are attributable to the OSAS [14]. A Swedish study has shown that the CPAP use ≥4 h/night was associated with a reduction of an MVA incidence [15]. However, no previous cohort study has been conducted to examine the association between OSA and the risk of injury related to hospitalization for MVAs. Therefore, a study is needed to estimate the risk of hospitalization for an MVA injury in patients with OSA in Taiwan. This is an exploratory study to investigate as towhether OSA and the usage of modafinil and methylphenidate, CPAP, and pharyngeal surgery were associated with the risk of hospitalization for an MVA injury. We thereby conducted this cohort study, using Taiwan’s nationwide database (the National Health Insurance Research Database [NHIRD]), to examine as to whether the OSA has been associated with the risk of hospitalization for an MVA injury, and the association between the usage of stimulants, CPAP, or pharyngeal surgery, and the risk of hospitalization for an MVA injury in the OSA patients.

## Methods

### Data sources

The National Health Insurance (NHI) Program was launched in Taiwan in 1995, and as of June 2009, included contracts with 97% of the medical providers with approximately 23 million beneficiaries, or more than 99% of the entire population [16, 17]. The NHIRD, which contains all the claims data of the beneficiaries, uses the International Classification of Diseases, 9th Revision, Clinical Modification (ICD-9-CM) codes to record the diagnoses [18]. The details of the program have been documented in previous studies [19–24].

A subset of the NHIRD, the Longitudinal Health Insurance Database (LHID) of one million randomized sampled population between 2000 and 2015, was used to study the association between OSA and the risk of hospitalization for MVA injury. The present study used the NHIRD to identify patients aged ≧ 20 with a diagnosis of OSA, based on the ICD-9-CM codes, 327.23, 780.51, 780.53, and 780.57. Patients with narcolepsy, ICD-9-CM code 347, were excluded. In Taiwan, the OSA diagnostic criteria is according to the Report of an American Academy of Sleep Medicine Task Force published in 1999, that is, the presence of sleep disordered breathing measured in an overnight sleep study combined with the presence of symptoms typical of the disorder, most notably excessive daytime sleepiness [25], during all the study period. Each enrolled patient in the OSA cohort was required to have received the procedure codes of polysomnography (PSG) within 1 year before or after the OSA code occurred, during 2000–2015. In one recent study, the accuracy of diagnosis of the OSA recorded in the NHIRD, has been validated as 99% [26].

### Study design and sampled participants

This study was of a population-based, matched-cohort design. Patients aged ≧ 20 with newly diagnosed OSA were selected from the LHID between January 1, 2000, and December 31, 2015. The patients with OSA before 2000 were excluded. In addition, the patients diagnosed with an injury related to MVAs, (ICD-9-CM codes: E810-E819) plus injuries codes (ICD-9-CM codes: 800.xx– 999.xx), before 2000, or before the first visit for OSA were also excluded (Additional file 1: Figure S1).

### Covariates

The covariates included sex, age groups (20–44, 45–64, and ≧ 65 years), geographical area of residence (north, center, south, and east of Taiwan), urbanization level of residence (levels 1 to 4), and monthly insured premiums (in New Taiwan Dollars [NT$]; < 18,000, 18,000-34,999, ≥35,000). A health insurance premium is an upfront payment made on behalf of an individual or family in order to keep their health insurance policy active, and the insured premiums for the NHI are based on the salary of the insured individuals, and calculated according to the NHI policy [27]. The urbanization level of residence was defined according to the population and various indicators of the level of development. Level 1 was defined as a population of > 1,250,000, and a specific designation as political, economic, cultural, and metropolitan development. Level 2 was defined as a population between 500,000 and 1,249,999, and as playing an important role in the politics, economy, and culture. Urbanization levels 3 and 4 were defined as a population between 149,999 and 499,999, and < 149,999, respectively.

Usage of psychostimulants, including modafinil or methylphenidate, and records of procedures of tonsillectomy, adenoidectomy, adenotonsillectomy, and vulopalatopharyngoplasty, were collected. The proportion of days covered (PDC) by medications within the entire follow-up period was divided into two categories (1–50%, 51–100%) to compare the levels of adherence to the drug regimens by the patients, and to correlate the adherence level with the incidence of hospitalization for MVA injury. The CPAP is not reimbursed in the NHI policy, therefore, we collected this record with the reference of one previous research. Briefly, among the patients diagnosed with OSA who used CPAP, an additional PSG would be performed within the first 6 months of the OSA diagnosis, for the titration of the CPAP. Therefore, we used the additional PSG records 6 months after the OSA diagnosis to define the cases that used CPAP [28].

The usage of hypnotic (Z-drugs and benzodiazepines), and other sedative medications such as antihistamines, antidepressants, and antipsychotics commonly used in Taiwan [29], were also analyzed as the covariates. The data of the defined daily dose (DDD) were obtained from the WHO Collaborating Centre for Drug Statistics Methodology (https://www.whocc. no/), and the duration of the usage of hypnotics, antidepressants, antipsychotics, and antihistamines was calculated by dividing the cumulative doses by the DDD of these medications.

### Comorbidity

Comorbidities were assessed using the Charlson Comorbidity Index (CCI), which categorizes the comorbidities using the ICD-9-CM codes, scores each comorbidity category, and combines all the scores to calculate a single comorbidity score, with different weights (Additional file 2: Table S1). A score of zero indicates that no comorbidities were found, and higher scores indicate higher comorbidity burdens [30–32]. The comorbidities including insomnia (ICD-9-CM codes: 307.4, 780.5 except 780.51, 780.53, 780.57), idiopathic hypersomnia (ICD-9-CM: 327.11, 327.12), circadian rhythm sleep disorder, shift work type (ICD-9-CM Code: 327.36), restless leg syndrome (RLS, ICD-9-CM code: 333.94), periodic leg movements disorder (PLMD; ICD-9-CM code: 327.51), anxiety disorders (ICD-9-CM codes: 300.x), depressive disorders (ICD-9-CM codes: 296.2x-296.3x, 300.4, 311), bipolar disorders (ICD-9-CM codes: 296.0, 296.4x-296.8x), psychotic disorders (ICD-9-CM codes: 295.xx, 297–298), alcohol-related disorders (ICD-9-CM codes: 303.x, 305.0x, V11.3; 291.x), and other substance-related disorders (ICD-9-CM codes: 304.0x, 304.1x, 304.2x, 304.3x, 304.5x, 304.6x, 304.7x, 304.8x, 304.9x, 305.0x, 305.1x, 305.2x, 305.3x, 305.4x, 305.5x, 305.6x, 305.8x, 305.9x, and 292.x) were also assessed in this study.

### Major outcomes

All of the study participants were followed from the index date until the suffering of hospitalization for an MVA injury (ICD-9-CM codes: E810-E819) plus injuries codes (ICD-9-CM codes: 800.xx– 999.xx, withdrawal from the NHI program, or the end of 2015. During this study, there were 6.17% participants missing from the study participants by withdrawal from the NHI program. The severity of injury was also analyzed in this study, and was classified as the Injury Severity Score (ISS) < 16 or ≧ 16, injury diagnosis, and the types were also recorded, since the ISS is an established medical score to assess the trauma severity, while patients with the ISS ≧ 16 was defined as a major trauma [33].

### Statistical analysis

All statistical analyses were performed using the SPSS for Windows, version 22.0 (IBM Corp., Armonk, NY). Χ^2^ and t tests were used to evaluate the distributions of the categorical and continuous variables, respectively, with a Fischer exact examination. The multivariate Cox proportional hazards regression analysis was used to determine the risk of the hospitalization for an MVA injury, and the results were presented as a hazard ratio (HR) with a 95% confidence interval (CI). The difference in the risk of hospitalization for an MVA injury, between the OSA cohort and the non-OAS control, was estimated using the Kaplan-Meier method with the log-rank test. A 2-tailed *p* value < 0.05 was considered to indicate the statistical significance.

## Results

### Sample characteristics

Table 1 shows the sex, age, comorbidities, urbanization, area of residence, monthly insured premiums of the OSA patients, and the controls. When compared to the controls, the OSA patients tended to have more insomnia, idiopathic hypersomnia, anxiety disorder, depressive disorder, bipolar disorder, psychotic disorder, alcohol-related disorders and other substance-related disorders. The OSA patients had higher CCI scores (CCI =1.8 ± 3.1 [range: 0–24] vs 1.0 ± 2.0 [range: 0–22], *p* < 0.001), and the OSA patients tended to have higher rates of living in the urbanization level 1 area, and in the north and middle of Taiwan, than the control groups. In addition, in the OSA patients, there were 70 (0.77%) individuals with insomnia (307.4 and 780.5X [excluding 780.51, 780.53, and 780.57]).

**Table 1:** Characteristics of study at the baseline

Variables	OSA		No OSA		*P*
	n	%	n	%	
Total	3025	25.0	9075	75.0	
Sex					0.999
Male	2030	67.1	6090	67.1	
Female	995	32.9	2985	32. 9	
Age (years)	56.1 ± 18.2		56.0 ± 18.0		0.662
Age group (years)					0.999
20–44	755	25.0	2265	25.0	
45–64	1065	35.2	3195	35.2	
≧65	1205	39.8	3615	39.8	
Insured premium (NT$)	20,124.3 ± 22,454.2		20,207.1 ± 22,579.8		0.861
Insured premium group (NT$)	α				0.910
< 18,000	2564	84.8	7661	84.4	
18,000-34,999	292	9.7	897	9.9	
≧35,000	169	5.6	517	5.7	
CCI	1.8 ± 3.1 (range: 0–24)		1.0 ± 2.0 (range: 0–22)		< 0.001
Comorbidities					
Insomnia	1875	62.0	3126	34.4	< 0.001
Restless leg syndrome	9	0.3	25	0.3	0.843
Idiopathic hypersomnia	21	0.7	6	0.1	< 0.001
Circadian rhythm sleep disorder, shift work type	2	0.1	0	0.0	0.062
Anxiety disorders	785	26.0	1024	11.3	< 0.001
Depressive disorders	998	33.0	1186	13.1	< 0.001
Bipolar disorders	371	12.3	690	7.6	< 0.001
Psychotic disorders	624	20.6	834	9.2	< 0.001
Periodic limb movements disorder	3	0.1	8	0.1	0.862
Alcohol-related disorders	216	7.1	485	5.3	< 0.001
Other substance-related disorders	397	13.1	679	7.5	< 0.001
Medications					
Hypnotics (Z-drugs and benzodiazepine)	2262	74.8	1986	21.9	< 0.001
Antihistamines	1264	41.8	3346	36.9	< 0.001
Antidepressants	1095	36.2	2131	23.5	< 0.001
Antipsychotics	1176	38.9	2510	27.7	< 0.001
Location					< 0.001
Northern Taiwan	1389	45.9	3756	41.4	
Middle Taiwan	900	29.8	2552	28.1	
Southern Taiwan	669	22.1	2234	24.6	
Eastern Taiwan	66	2.2	502	5.5	
Outlets islands	1	0.03	31	0.3	
Urbanization level					< 0.001
1 (The highest)	1528	50.5	3130	34.5	
2	1057	34.9	3970	43.8	
3	189	6.3	696	7.7	
4 (The lowest)	251	8.3	1279	14.1	
Level of care					< 0.001
Medical center	1706	56.4	3191	35.2	
Regional hospital	910	30.1	3182	35.1	
Local hospital	409	13.5	2702	29.8	

*OSA* obstructive sleep apnea, *P* Chi-square/Fisher exact test on category variables and t-test on continue variables, *NT$* New Taiwan Dollars, *CCI* Charlson comorbidity index

### Kaplan-Meier model for the cumulative risk of psychiatric disorders

**Fig. 1:**
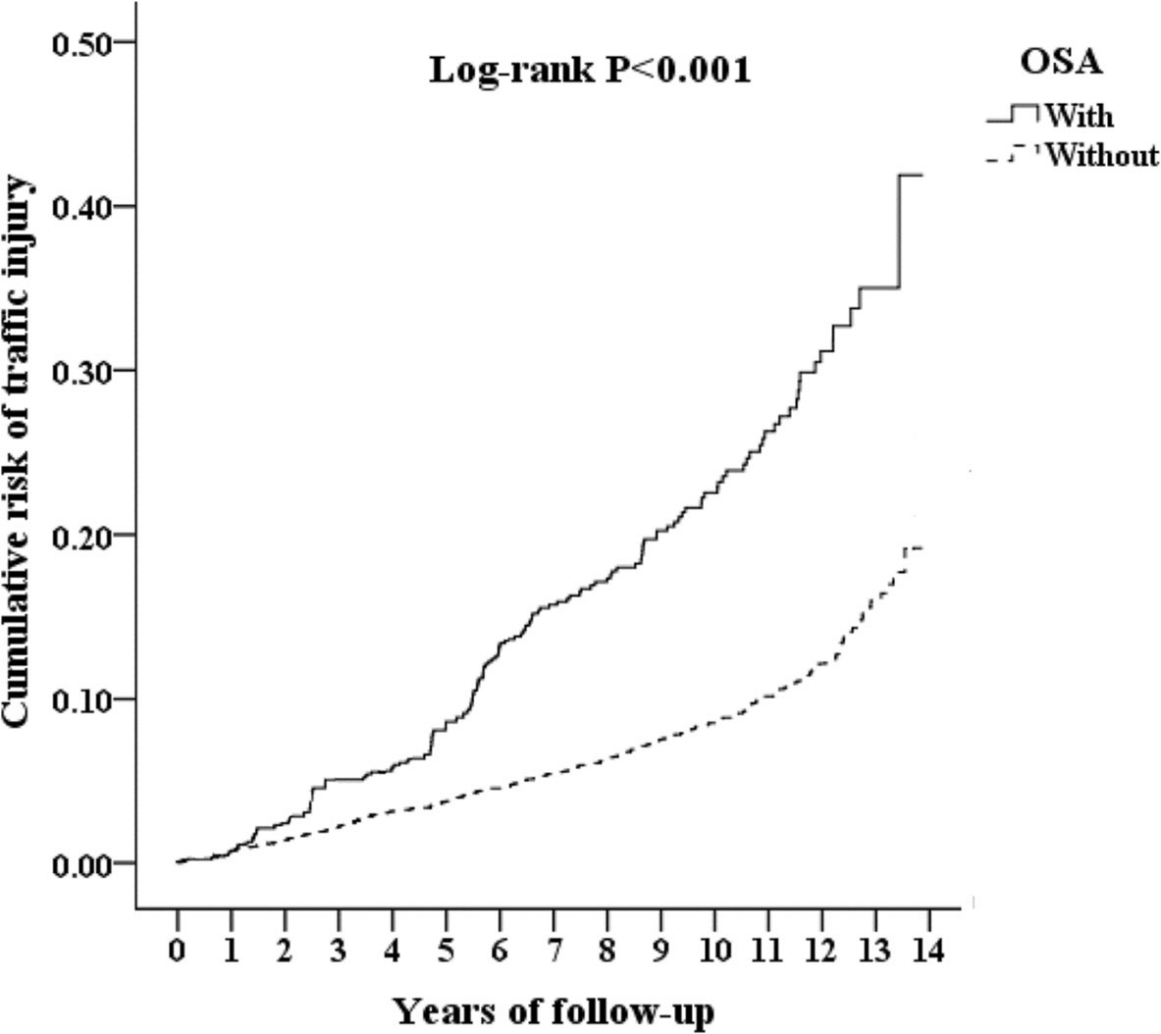
Kaplan-Meier for cumulative incidence of hospitalization for the hospitalization for MVA injury among aged 20 and over stratified by OSA with log-rank test (MVA = motor vehicle accident)

The cumulative incidence of the hospitalization for an MVA injury in the OSA patients and control groups, and the differences between the two groups were significant (log-rank test, *p* < 0.001, Fig. 1).

### Hazard ratios analysis of the hospitalization for MVA injury and mortality in OSA patients

In this study, 172 (5.7%, or 575.3 per 100,000 person-year) in the OSA cohort, and 358 (3.97%, 372.0 per 100,000 person-year) suffered from hospitalization for an MVA injury in the non-OSA controls (*p* < 0.001, Table 2). Table 2 also shows the Cox regression analysis of the factors associated with the risk of an MVA injury. The crude HR was 2.35 (95% CI: 1.96–2.81, *p* < 0.001), after adjusting for age, sex, CCI scores, geographical area of residence, urbanization level of residence, and monthly income, the adjusted HR was 2.18(95% CI: 1.79–2.64, *p* < 0.001). For the OSA patients with comorbidities such as insomnia, anxiety disorder, depressive disorder, bipolar disorder, psychotic disorder, alcohol-related disorders and other substance-related disorders were associated with the risk of hospitalization for MVA injury, in comparison to those without these comorbidities. The OSA patients with medications, such as hypnotic (Z-drugs and benzodiazepines) and other sedative medications such as antihistamines, antidepressants, and antipsychotics, were associated with the risk of hospitalization for an MVA injury, in comparison to those without these medications. Furthermore, the risk of MVA hospitalization would have increased 57% at every score of CCI.

**Table 2:** Distribution and hazard ratio of hospitalization for motor vehicle accident injury in OSA patients

OSA vs No OSA (N)	3025	9075
MVA injury^*^ N (%)	172 (5.7%)	358 (3.97%)
per 10^5^ person-year	575.3	392.0
Crude HR *(reference: non-OSA)*	2.35 (95% CI: 1.96–2.81) ^***^	
Adjusted HR *(reference: non-OSA)*	2.18 (95% CI: 1.79–2.64) ^***^	
Male *(reference: female)*, adjusted HR	1.48 (95% CI: 1.18–1.71) ^***^	
CCI, adjusted HR	1.57 (95% CI: 1.55–1.59) ^***^	
Comorbidities *(reference: without)*, adjusted HR		
Insomnia	2.96 (95%CI: 1.43–5.33) ^***^	
Idiopathic hypersomnia	18.22 (95%CI 0.45–167.21)	
Circadian rhythm sleep disorder, shift work type	No MVA injury events	
Anxiety disorders	5.11 (95%CI: 3.98–7.66) ^***^	
Depressive disorders	4.35 (95%CI: 2.06–6.53) ^***^	
Bipolar disorders	3.38 (95%CI: 2.11–4.79) ^***^	
Psychotic disorders	2.64 (95%CI: 1.29–3.86) ^***^	
Restless leg syndrome	No MVA injury events	
Periodic limb movement disorder	No MVA injury events	
Alcohol-related disorders	5.71 (95%CI: 2.63–8.82) ^***^	
Other substance-related disorders	3.59 (95%CI: 1.11–4.94) ^*^	
Medications *(reference: without)*, adjusted HR		
Hypnotics (Z-drugs and benzodiazepine)	2.31 (95%CI: 1.84–2.98) ^***^	
Antihistamines	1.30 (95%CI: 1.01–1.62) ^*^	
Antidepressants	1.64 (95%CI: 1.24–2.13) ^**^	
Antipsychotics	1.81 (95%CI: 1.31–2.20) ^***^	

*OSA* obstructive sleep apnea, *MVA* motor vehicle accidents, *HR* hazard ratio, *CCI* Charlson comorbidity index; Adjusted for the variables listed in Table 1

^*^ *P* < 0.05, ^**^ *P* < 0.01, ^***^ *P* < 0.001

In this study, 10 (0.33%, or 33.4 per 100,000 person-year) in the OSA cohort, and 4 (0.04%, 4.2 per 100,000 person-year) in the non-OSA controls, suffered from an MVA injury mortality (*p* < 0.05). The crude HR was 5.11 (95% CI: 1.59–16.35, *p* < 0.001), after adjusting for age, sex, CCI scores, geographical area of residence, urbanization level of residence, and monthly income, the adjusted HR was 2.25 (95% CI: 1.57–2.94, *p* < 0.001). In addition, in this study, 200 (6.61%, or 669.0 per 100,000 person-year) in the OSA cohort, and 358 (3.97%, 790.7 per 100,000 person-year) in the non-OSA controls, suffered from the all-cause mortality (*p* < 0.05). The crude HR was 0.86 (95% CI: 0.75–1.04), after adjusting for age, sex, CCI scores, geographical area of residence, urbanization level of residence, and monthly income, the adjusted HR was 1.02 (95% CI: 0.88–1.59) (Additional file 3: Table S2).

### Subgroup analysis of with stratification by factors of comorbidities and medications and the risk of hospitalization for MVA injury

Additional file 4: Table S3 depicts that the subgroup analysis of the stratification by factors of the comorbidities and medications and the risk of hospitalization for an MVA injury. The OSA patients, in comparison to the non-OSA controls, were associated with the risk of hospitalization for an MVA injury, no matter with or without insomnia, anxiety disorders, depressive disorders, bipolar disorders, psychotic disorders, and other substance-related disorders.

### Hazard ratio analysis of the hospitalization for MVA injury in different types and severity in OSA patients

The OSA cohort was associated with an increased risk in injury diagnosis as being fracture, dislocation, intracranial/internal injury, open wound, crushing, nerves and spinal cord injury, and other injuries, associated with injury types as drivers of motor vehicles, and other types, when compared to the control group. The OSA cohort was associated with the overall increased risk of the hospitalization for an MVA injury both in the ISS score < or ≧ 16 (Table 3).

**Table 3:** Hazard Ratios in injury diagnosis, types, and severity with significant findings, after Cox regression model analysis

Injury subgroup	OSA			No OSA			Adjusted HR	95% CI	95% CI
	Event	PYs	per 10^5^ PYs	Event	PYs	per 10^5^ PYs			
Total	172	29,895.12	575.34	358	96,242.00	371.98	2.18 ^***^	1.79	2.64
Injury diagnosis									
Fracture	48	29,895.12	160.56	163	96,242.00	169.36	1.33 ^*^	1.15	1.69
Dislocation	6	29,895.12	20.07	8	96,242.00	8.31	3.39 ^***^	2.80	4.12
Intracranial / internal injury	30	29,895.12	100.35	106	96,242.00	110.14	1.28 ^*^	1.03	1.77
Open wound	9	29,895.12	30.11	27	96,242.00	28.05	1.51 ^**^	1.19	1.89
Crushing	3	29,895.12	10.04	1	96,242.00	1.04	13.58 ^***^	11.27	16.39
Injury to nerves and spinal cord	1	29,895.12	3.35	2	96,242.00	2.08	2.27 ^***^	1.86	2.78
Other injury	65	29,895.12	217.43	14	96,242.00	14.55	21.03 ^***^	17.43	25.35
Injury types									
Driver of motor vehicle	5	29,895.12	16.73	11	96,242.00	11.43	2.07 ^***^	1.70	2.56
Others	53	29,895.12	177.29	87	96,242.00	90.40	2.76 ^***^	2.25	3.30
MVA injury severity									
ISS < 16	98	29,895.12	327.81	224	96,242.00	232.75	1.98 ^***^	1.61	2.40
ISS ≧16	74	29,895.12	247.53	134	96,242.00	139.23	2.51 ^***^	2.02	3.13

*PYs* Person-years, *Adjusted HR* Adjusted Hazard ratio, *CI* confidence interval, *MVA* motor vehicle accidents

^*^ *P* < 0.05, ^**^P < 0.01, ^***^P < 0.001

### Modafinil and methylphenidate and the risk of the hospitalization for MVA injury in OSA patients

Table 4 depicts the modafinil and methylphenidate usage and the risk of hospitalization for the MVA injury of the OSA cohort. In our study, modafinil or methylphenidate usage was associated with a nearly 20% decreased risk of the overall hospitalization for MVA injury. In general, the higher the PDC of these medications was used, the lower the risk of hospitalization for an MVA injury was found. However, for the patients of medication PDC of 51—100%, the usage of these medications was not associated with a decreased risk of injury in the patients with ISS ≧ 16. Neither the CPAP nor pharyngeal surgery was associated with a decreased risk of hospitalization of an MVA injury. The distributions of stimulants and theCPAP/pharyngeal surgery among the OSA patients were shown in Additional file 5: Table S4 and Additional file 6: Table S5, respectively. The numbers of modafinil, methylphenidate, and pharyngeal surgery used in the OSA patients are as shown in the supplementary tables (Additional file 5: Table S4 and Additional file 6: Table S5). Additional file 7: Table S6 shows that both individual and joint treatment of the CPAP and stimulants were associated with a lower risk of MVA injury.

**Table 4:** Factors of traffic injury by using Cox regression among OSA patients in different model

	Total			ISS < 16			ISS ≧ 16		
	Adjusted HR	95% CI	95% CI	Adjusted HR	95% CI	95% CI	Adjusted HR	95% CI	95% CI
Either modafinil or methylphenidate (*N* = 1690)	0.82 ^***^	0.70	0.83	0.78 ^***^	0.68	0.80	0.89	0.74	1.02
PDC 1—50% (*N* = 1264)	0.87 ^**^	0.71	0.88	0.82 ^***^	0.69	0.86	0.95	0.76	1.09
PDC 51—100% (*N* = 426)	0.78 ^***^	0.69	0.79	0.73 ^***^	0.67	0.76	0.85 ^*^	0.73	0.97
Modafinil (*N* = 933)	0.83 ^***^	0.71	0.84	0.78 ^***^	0.69	0.82	0.90	0.76	1.05
PDC 1—50% (*N* = 685)	0.89 ^**^	0.73	0.90	0.84 ^***^	0.71	0.87	0.97	0.78	1.11
PDC 51—100% (*N* = 248)	0.78 ^**^	0.70	0.79	0.73 ^***^	0.68	0.77	0.85	0.73	1.01
Methylphenidate (*N* = 893)	0.82 ^***^	0.69	0.92	0.77 ^***^	0.67	0.79	0.89	0.73	1.01
PDC 1—50% (*N* = 680)	0.85 ^***^	0.70	0.87	0.80 ^***^	0.68	0.85	0.93	0.73	1.08
PDC 51—100% (*N* = 213)	0.78 ^***^	0.69	0.80	0.73 ^***^	0.67	0.76	0.85 ^*^	0.72	0.96
Modafinil only (*N* = 797)	0.88 ^*^	0.82	0.96	0.94 ^*^	0.79	0.93	0.96	0.86	1.17
PDC 1—50% (*N* = 584)	0.90	0.82	1.02	0.85 ^*^	0.80	0.99	1.00	0.86	1.24
PDC 51—100% (N = 213)	0.85 ^*^	0.81	0.93	0.80 ^**^	0.78	0.90	0.93 ^*^	0.85	0.99
Methylphenidate only (*N* = 757)	0.84 ^*^	0.78	0.90	0.77 ^***^	0.76	0.88	0.92	0.82	1.12
PDC 1—50% (*N* = 579)	0.86 ^*^	0.79	0.93	0.81 ^***^	0.76	0.89	0.94	0.83	1.14
PDC 50—100% (*N* = 178)	0.83 ^**^	0.77	0.89	0.78 ^***^	0.74	0.86	0.90 ^*^	0.81	0.96
Modafinil & Methylphenidate (*N* = 136)	0.79 ^***^	0.64	0.85	0.74 ^***^	0.62	0.83	0.86	0.67	1.05
PDC 1—50% (*N* = 101)	0.81 ^***^	0.65	0.86	0.77 ^***^	0.63	0.84	0.88	0.69	1.06
PDC 50—100% (*N* = 35)	0.78 ^***^	0.63	0.79	0.70 ^***^	0.61	0.74	0.81 ^*^	0.66	0.94

*HR* hazard ratio, *CI* confidence interval, *Adjusted HR* Adjusted variables listed in Table 1

^*^ *P* < 0.05, ^**^ *P* < 0.01, ^***^ *P* < 0.001

## Discussion

### Association between OSA and the risk of the hospitalization for MVA injury

In this study, we have examined the association between the OSA and the risk of an MVA injury. After adjusting covariates, the adjusted HR was2.18 for the OSA cohort (95% CI: 1.79–2.64, *p* < 0.001) when compared with the control group. In other words, the OSA cohort had a 2.3-fold risk of developing an MVA injury. The Kaplan-Meier analysis revealed that the study subjects had a significantly higher 15-year hospitalization for the MVA incidence than the controls (log rank, *p* < 0.001). Furthermore, in this study, the OSA was associated with the increased risk of hospitalization for MVA injury in the severity of the ISS score < or ≧ 16. Usage of modafinil and methylphenidate was associated with a nearly 20% decreased risk of the hospitalization for an MVA injury in the OSA cohort. This study is distinct since we found the association between OSA and the hospitalization for an MVA injury, in comparison to the one previous study about the association between OSA and the crashes from the MVA [10, 11]. To the best of our knowledge, this is the first study that depicts the decreased risk of the hospitalization for an MVA injury risk in the OSA patients with modafinil or methylphenidate usage, from a nationwide, claims database in Taiwan.

### Comparison of this study to previous literatures

In comparison to previous studies on the association between OSA and MVA [13–15], this study was distinct in the following points: first, this is a cohort study from a larger nationwide database, in comparison to case control or cross-sectional studies. Second, we have examined the effects of the stimulants on the risk of the hospitalization for an MVA injury in the OSA patients. Previous studies have found that stimulants would improve the OSA patients driving performance [34, 35]. Previous articles of systematic reviews and meta-analysis have reported the association between OSA and the motor vehicle crashes, and the effects of CPAP therapy [10, 36]. In our study, we found that modafinil and methylphenidate usage was associated with a lower risk of the overall hospitalization for an MVA injury, not just motor vehicle crashes. In general, the higher the PDC of these medications used, the lower the risk of hospitalization for the MVA injury was found. A larger clinical trial for the OSA patients with modafinil or methylphenidate might well be needed to confirm this association.

### Possible mechanisms for the increased risk of the hospitalization for MVA injury in OSA patients

Patients with OSA, EDS, or hypersomnolence, are associated with the risk of the hospitalization for an MVA injury, since the OSA with EDS would impair the safety of driving, while the driving simulator technology declines in driving performance due to the EEG-defined microsleeps, using the driving simulated technology [37, 38]. In this study, insomnia, anxiety disorder, depressive disorder, bipolar disorder, psychotic disorder, alcohol-related disorders, other substance-related disorders, and several medications such as hypnotics, antihistamines, antidepressants, and antipsychotics, were associated with hospitalization for an MVA injury. The impact of EDS from the comorbidities and concomitant medications needed to be warranted. The idiopathic hypersomnia was not associated with the risk of hospitalization for the MVA injury.

The OSA could also impair the cognitive performance in attention, memory, and fronto-executive functioning [39, 40], and this could also contribute to the risk-proneness in the OSA patients. Previous studies have reported that the usage of stimulants could improve wakefulness and cognitive performance in the OSA patients [41, 42], and our study also concords with these findings by showing a lower risk of the hospitalization for an MVA injury in the OSA patients who received modafinil or methylphenidate.

### CPAP and pharyngeal surgery and the risk of the hospitalization for MVA injury in OSA patients

As the CPAP treatment is not reimbursed by the NHI policy, we therefore could only use indirect methods to confirm the usage of the CPAP treatment as aforementioned. The lower usage of CPAP might be related to the findings that the acceptance of CPAP is low in Taiwan [43, 44]. The usage of oral appliances, such as the mandibular advancement devices, is not reimbursed by the NHI, therefore, we could not evaluate the effects of these appliances on the hospitalization for an MVA injury in the OSA cohort.

### Strengths of this study

The present study has several strengths: First, we used the NHIRD with a large sample size in this study, and, as aforementioned, in one recent study, the accuracy of diagnosis of OSA recorded in the NHIRD, has been validated as 99% [26]. Second, a 1:3 sex-, age-, index date-matched comparator group was enrolled in this study. Third, we have assessed as to whether the usage of modafinil or methylphenidate alters the risk of hospitalization for an MVA injury in OSA. Fourth, we excluded patients who were comorbid with narcolepsy, and thus, we could evaluate the effects of modafinil or methylphenidate only for the OSA patients.

### Limitations of this study

The present study has several limitations that warrant consideration. First, similar to previous studies using the NHIRD on the OSA as aforementioned, not all of the data were recorded in the NHIRD, and we were unable to evaluate the severity, weakness severity, laboratory parameters, or lung function examinations in the apnea patients. However, one recent study has validated that the accuracy of diagnosis of the OSA recorded in the NHIRD was 99%, as aforementioned [26]. Second, other factors, such as the body mass index, genetic, psychosocial, environmental factors, and lifestyle, were not included in the dataset. For example, we have analyzed the circadian rhythm sleep disorder, shift work type instead of the lifestyle as shift work. Third, the lack of data on the severity of the OSA would limit the generalization of the results of this study. Although the diagnosis of the OSA requires overnight PSG to detect the frequency of apneic and hypopneic events [45, 46]. the NHIRD does not contain the records of overnight oximetry screening prior to deciding about PSG, which may produce a 17% false negative rate [47]. Fourth, the NHI program started in 1995, but in this study, the NHIRD we used, contained only a database of 15 years. We would like to highly recommend that a longer follow-up study is needed in the future. Fifth, there were no direct records of the CPAP in the NHIRD, the usage of CPAP therapy is crudely based on whether an additional PSG was requested, within the first 6 months after the OSA diagnosis, However, one study about the acceptance and adherence for the CPAP found that the continuous (nightly) use of the CPAP was around 66.7—74.3%, which were higher than the discontinuous (irregular) users, or, in the OSA patients who were prescribed this treatment [48]. Sixth, psychostimulants for other sleep problems such as narcolepsy and EDS might well be associated with the decreased risk of hospitalization for MVA injury, however, we have excluded the diagnosis of narcolepsy from the enrollees. Seventh, the usage of some substances such as alcohol, marijuana and cocaine were associated with EDS [49], and thus could be associated with the risk of hospitalization for an MVA injury, however, the records of usage of alcohol, marijuana, cocaine or other psychoactive substances were not included in the NHIRD. We therefore analyzed the alcohol- or other substance-related disorders in the present study and found that these disorders were associated the increased risk of hospitalization for an MVA injury, in comparison to those without these comorbidities. Finally, in such a claims database study, the records might not contain all the records. In addition, these records might not reflect the fact that many different healthcare professionals have been involved in patient care, so the measurement of risk factors and outcomes throughout the database would probably be less accurate and consistent than that achieved with a prospective cohort study design.

## Conclusion

This study found a nearly 2.2-fold higher risk of hospitalization for an MVA injury in the untreated OSA patients. Furthermore, modafinil and methylphenidate usage was associated with a nearly 20% decreased risk of the overall hospitalization for an MVA injury in patients with OSA. These findings strongly suggest that clinicians should now provide a careful follow-up and medical treatment for these patients.

## Supplementary information

**Additional file 1: Figure S1.** The flowchart of study sample selection from National Health Insurance Research Database in Taiwan.

**Additional file 2: Table S1.** Diagnosis and weights in the Charlson Comorbidity Index.

**Additional file 3: Table S2.** Distribution and hazard ratio of mortality for motor vehicle accident injury and all-cause mortality in OSA patients.

**Additional file 4: Table S3.** Subgroup analysis of with stratification by factors of comorbidities and medications and the risk of hospitalization for MVA injury.

**Additional file 5: Table S4.** Distribution of medication among OSA patients.

**Additional file 6: Table S5.** Distribution of Treatment surgery.

**Additional file 7: Table S6.** Factors of traffic injury by using Cox regression among OSA patients in different treatments.

## Data Availability

Data are available from the National Health Insurance Research Database (NHIRD) published by Taiwan National Health Insurance (NHI) Bureau. Due to legal restrictions imposed by the government of Taiwan in relation to the “Personal Information Protection Act”, data cannot be made publicly available. Requests for data can be sent as a formal proposal to the NHIRD (http://nhird.nhri.org.tw).

## Abbreviations

CCI: Charlson Comorbidity Index
CI: Confidence interval
CPAP: Continuous positive airway pressure
EDS: Excessive daytime sleepiness
HR: Hazard ratio
ICD-9-CM: International Classification of Diseases, 9th Revision, Clinical Modification
ISS: Injury Severity Score
LHID: Longitudinal Health Insurance Database
MVA: Motor vehicle accident
NHI: National Health Insurance
NHIRD: National Health Insurance Research Database
NT$: New Taiwan Dollars
OSA: Obstructive sleep apnea
PDC: Proportion of days covered
PSG: Polysomnography
