# Supplementary material for: Stimulants associated with reduced risk of hospitalization for motor vehicle accident injury in patients with obstructive sleep apnea-a nationwide cohort study
Source: BMC Pulm Med. 2020 Feb 3;20:28. doi: 10.1186/s12890-019-1041-1 (PMC6998364; doi:10.1186/s12890-019-1041-1)
Supplement: Supplementary file 1 — Additional file 1: Figure S1. The flowchart of study sample selection from National Health Insurance Research Database in Taiwan. [file 12890_2019_1041_MOESM1_ESM.docx]

| Outpatient and inpatient of Longitudinal Health Insurance Database (LHID) in 2000-2013 in Taiwan  **26,769,418 events; 989,753 individuals** | | | | | | | | | | | | |
| --- | --- | --- | --- | --- | --- | --- | --- | --- | --- | --- | --- | --- |
|  | |  | |  | | | |  | |  |  |  |
| Inclusion criteria  OSAS with PSG 1 year before or 1 year after the index date  **4,297 individuals** | | | | |  | | |  | |  |  |  |
|  | |  | |  | | | Exclusion criteria   1. OSAS before index date 2. Injury / traffic injury before tracking 3. Age < 20 years 4. Gender unknown   **1,272 individuals** | | | | |  |
|  | |  | |  | | | |  | |  |  |  |
|  | |  | |  | | 1. Without sleep disorders in study period 2. The same exclusion criteria of study cohort 3. 3-fold propensity score matching by gender, age, and index year | | | | | |  |
|  | |  | |  | | | |  | |  |  |  |
|  | With OSAS (Study cohort)  **3,025** **individuals** | | | | | | |  | Without OSAS (Comparison cohort)  **9,075 individuals** | | |  |
|  | |  | Tracking endpoint (Dec 31, 2013) | | | | | | | |  |  |
|  | **172 individuals** with traffic injury | | | | | | |  | **358 individuals** with traffic injury | | |  |

**Figure 1.** The flowchart of study sample selection from National Health Insurance Research Database in Taiwan
