# Supplementary material for: Stimulants associated with reduced risk of hospitalization for motor vehicle accident injury in patients with obstructive sleep apnea-a nationwide cohort study
Source: BMC Pulm Med. 2020 Feb 3;20:28. doi: 10.1186/s12890-019-1041-1 (PMC6998364; doi:10.1186/s12890-019-1041-1)
Supplement: Supplementary file 2 — Additional file 2: Table S1. Diagnosis and weights in the Charlson Comorbidity Index. [file 12890_2019_1041_MOESM2_ESM.docx]

| **Table S1. Diagnosis and weights in the Charlson Comorbidity Index** | | |
| --- | --- | --- |
| **Diagnosis** | **ICD-9-CM codes** | **Score** |
| **None of the following diagnosis** |  | 0 |
| **Myocardial infarction** | 410、412 | 1 |
| **Congestive heart failure** | 428 | 1 |
| **Peripheral vascular** | 441、443.9、785.4、V43.4、Procedure 38.48 | 1 |
| **Cerebrovascular disease** | 430-438 | 1 |
| **Dementia** | 290 | 1 |
| **Chronic pulmonary disease** | 490-496、500-505、506.4 | 1 |
| **Rheumatologic disease** | 710.0-710.1、710.4、  714.0-714.2、714.81、725 | 1 |
| **Peptic ulcer disease** | 531-534 | 1 |
| **Mild liver disease** | 571.2、571.4-571.6 | 1 |
| **Diabetes mellitus** | 250.0-250.3、250.7 | 1 |
| **Diabetes with chronic complication** | 250.4-250.6 | 2 |
| **Hemiplegia or paraplegia** | 342、344.1 | 2 |
| **Renal disease** | 582、583.0-583.7、585-586、588 | 2 |
| **Malignancy, including leukemia and lymphoma** | 140-172、174-194、195.0-195.8、200-208 | 3 |
| **Moderate or server liver disease** | 572.2-572.8、456.0-456.21 | 3 |
| **Metastatic solid tumor** | 196-198、199.0-199.1 | 6 |
| **Acquired Immunodeficiency syndrome (AIDS)** | 042-044 | 6 |

**Adapted from [30-32]**
