# Supplementary material for: Stimulants associated with reduced risk of hospitalization for motor vehicle accident injury in patients with obstructive sleep apnea-a nationwide cohort study
Source: BMC Pulm Med. 2020 Feb 3;20:28. doi: 10.1186/s12890-019-1041-1 (PMC6998364; doi:10.1186/s12890-019-1041-1)
Supplement: Supplementary file 3 — Additional file 3: Table S2. Distribution and hazard ratio of mortality for motor vehicle accident injury and all-cause mortality in OSA patients. [file 12890_2019_1041_MOESM3_ESM.doc]

| **Table S2. Distribution and hazard ratio of mortality for motor vehicle accident injury and all-cause mortality in OSA patients** | | |
| --- | --- | --- |
| **OSA Non-OSA** | | |
|  | 3,025 | 9,075 |
| **MVA injury mortality* N (%)** | 10 (0.33%) | 4 (0.04%) |
| per 10^5^ person-year | 33.4 | 4.2 |
| Crude HR *(reference: non-OSA)* | 5.11 (95% CI: 1.59-16.35) ^***^ | |
| Adjusted HR *(reference: non-OSA)* | 2.25 (95% CI: 1.57-2.94) ^***^ | |
| **All-Caused mortality* N (%)** | 200 (6.61%) | 761 (8.39%) |
| per 10^5^ person-year | 669.0 | 790.7 |
| Crude HR *(reference: non-OSA)* | 0.86 (95% CI: 0.75-1.04) | |
| Adjusted HR *(reference: non-OSA)* | 1.02 (95% CI: 0.88-1.59) | |

**OSA: obstructive sleep apnea; MVA: motor vehicle accidents; HR: hazard ratio;**

**CCI: Charlson comorbidity index; Adjusted for the variables listed in Table 1**

**^*^ P < 0.05, ^**^ P < 0.01, ^***^ P < 0.001**
