# Supplementary material for: Stimulants associated with reduced risk of hospitalization for motor vehicle accident injury in patients with obstructive sleep apnea-a nationwide cohort study
Source: BMC Pulm Med. 2020 Feb 3;20:28. doi: 10.1186/s12890-019-1041-1 (PMC6998364; doi:10.1186/s12890-019-1041-1)
Supplement: Supplementary file 4 — Additional file 4: Table S3. Subgroup analysis of with stratification by factors of comorbidities and medications and the risk of hospitalization for MVA injury. [file 12890_2019_1041_MOESM4_ESM.doc]

| **Table S3. Subgroup analysis of with stratification by factors of comorbidities and medications and the risk of hospitalization for MVA injury** | | |
| --- | --- | --- |
| **Comorbidities** |  | **adjusted HR** |
| **Insomnia** | **Yes** | 3.15 (95%CI: 1.57-5.72) *** |
|  | **No** | 2.88 (95%CI: 1.31-4.86) *** |
| **Idiopathic hypersomnia** | **Yes** | 24.95 (95%CI: 0.59-289.15) |
|  | **No** | 11.12 (95%CI: 0.12-99.11) |
| **Circadian rhythm sleep disorder, shift work type** | **Yes** | No MVA injury events |
|  | **No** | No MVA injury events |
| **Anxiety disorders** | **Yes** | 5.76 (95%CI: 4.13-8.15) *** |
|  | **No** | 4.82 (95%CI: 3.50-7.42) *** |
| **Depressive disorders** | **Yes** | 4.52 (95%CI: 2.17-6.81) *** |
|  | **No** | 4.11 (95%CI: 2.00-6.24) *** |
| **Bipolar disorders** | **Yes** | 3.97 (95%CI: 2.86-5.01) *** |
|  | **No** | 3.12 (95%CI: 1.64-4.52) *** |
| **Psychotic disorders** | **Yes** | 2.85 (95%CI: 1.46-3.98) *** |
|  | **No** | 2.01 (95%CI: 1.13-3.35) ** |
| **Restless leg syndrome** | **Yes** | No MVA injury events |
|  | **No** | No MVA injury events |
| **Periodic limb movement disorder** | **Yes** | No MVA injury events |
|  | **No** | No MVA injury events |
| **Alcohol-related disorders** | **Yes** | 8.98 (95%CI: 3.45-15.767) *** |
|  | **No** | 3.12 (95%CI: 0.88-5.97) |
| **Other substance-related disorders** | **Yes** | 3.69 (95%CI: 1.27-5.11) *** |
|  | **No** | 3.42 (95%CI: 1.06-4.85) * |
| **Hypnotics (Z-drugs and benzodiazepine)** | **Yes** | 2.75 (95%CI: 1.89-3.75) *** |
|  | **No** | 2.01 (95%CI: 1.45-2.70) *** |
| **Antihistamines** | **Yes** | 1.42 (95%CI: 1.06-1.75) * |
|  | **No** | 1.28 (95%CI: 0.92-1.58) |
| **Antidepressants** | **Yes** | 1.72 (95%CI: 1.38-2.53) *** |
|  | **No** | 1.55 (95%CI: 1.09-2.06) * |
| **Antipsychotics** | **Yes** | 2.15 (95%CI: 1.44-2.68) *** |
|  | **No** | 1.69 (95%CI: 1.07-2.05) * |

**OSA: obstructive sleep apnea; MVA: motor vehicle injury; HR: hazard ratio; Adjusted for the variables listed in Table 1**

**^*^ P < 0.05, ^**^ P < 0.01, ^***^ P < 0.001**
