# Supplementary material for: Stimulants associated with reduced risk of hospitalization for motor vehicle accident injury in patients with obstructive sleep apnea-a nationwide cohort study
Source: BMC Pulm Med. 2020 Feb 3;20:28. doi: 10.1186/s12890-019-1041-1 (PMC6998364; doi:10.1186/s12890-019-1041-1)
Supplement: Supplementary file 5 — Additional file 5: Table S4. Distribution of medication among OSA patients. [file 12890_2019_1041_MOESM5_ESM.doc]

| **Table S4. Distribution of medication among OSA patients** | | |
| --- | --- | --- |
| **Medication subgroup** | **n** | **%** |
| **Total** | 3,025 |  |
| **Modafinil** |  |  |
| Without (PDC 0%) | 2,092 | 69.16 |
| With | 933 | 30.84 |
| PDC 1%-50% | 685 | 22.64 |
| PDC 51%-100% | 248 | 8.20 |
| **Methylphenidate** |  |  |
| Without (PDC 0%) | 2,132 | 70.48 |
| With | 893 | 29.52 |
| PDC 1%-50% | 680 | 22.48 |
| PDC 51%-100% | 213 | 7.04 |
| **Medication** |  |  |
| Without (PDC 0%) | 1,335 | 44.13 |
| Modafinil only | 797 | 26.35 |
| PDC 1%-50% | 584 | 19.31 |
| PDC 51%-100% | 213 | 7.04 |
| Methylphenidate only | 757 | 25.02 |
| PDC 1%-50% | 579 | 19.14 |
| PDC 51%-100% | 178 | 5.88 |
| Modafinil & Methylphenidate | 136 | 4.50 |
| PDC 1%-50% | 101 | 3.34 |
| PDC 51%-100% | 35 | 1.16 |
| **OSA = obstructive sleep apnea; PDC = the proportion of days covered** | | |
