# Supplementary material for: Stimulants associated with reduced risk of hospitalization for motor vehicle accident injury in patients with obstructive sleep apnea-a nationwide cohort study
Source: BMC Pulm Med. 2020 Feb 3;20:28. doi: 10.1186/s12890-019-1041-1 (PMC6998364; doi:10.1186/s12890-019-1041-1)
Supplement: Supplementary file 6 — Additional file 6: Table S5. Distribution of Treatment surgery. [file 12890_2019_1041_MOESM6_ESM.doc]

| **Table S5. Distribution of Treatment surgery** | | |
| --- | --- | --- |
|  | **OSA** | |
| **Treatment surgery** |  | **%** |
| **Total** | 3,025 | 25.00 |
| **CPAP** |  |  |
| Without | 2,919 | 96.50 |
| With | 106 | 3.50 |
| **Pharyngeal surgery** |  |  |
| Without | 2,891 | 95.57 |
| With | 134 | 4.43 |
| **Tonsillectomy/Adenoidectomy/Adenotonsillectomy** |  |  |
| Without | 2,874 | 95.01 |
| With | 151 | 4.99 |
| **Uvulopalatopharyngoplasty** |  |  |
| Without | 2,824 | 93.36 |
| With | 201 | 6.64 |
| **Treatment surgery** |  |  |
| Without | 2,718 | 89.85 |
| CPAP only | 106 | 3.50 |
| Pharyngeal surgery only | 201 | 6.64 |
| CPAP & Pharyngeal surgery | 0 | 0.00 |
| **Treatment surgery** |  |  |
| Without | 2,718 | 89.85 |
| CPAP only | 106 | 3.50 |
| Tonsillectomy / Adenoidectomy / Adenotonsillectomy only | 50 | 1.65 |
| Uvulopalatopharyngoplasty only | 67 | 2.21 |
| CPAP and Tonsillectomy / Adenoidectomy / Adenotonsillectomy | 0 | 0.00 |
| CPAP and Uvulopalatopharyngoplasty | 0 | 0.00 |
| Tonsillectomy / Adenoidectomy / Adenotonsillectomy and Uvulopalatopharyngoplasty | 84 | 2.78 |
| CPAP, Tonsillectomy / Adenoidectomy / Adenotonsillectomy, and Uvulopalatopharyngoplasty | 0 | 0.00 |

**OSA= obstructive sleep apnea, CPAP = continuous pressure airway pressure**
