# Supplementary material for: Stimulants associated with reduced risk of hospitalization for motor vehicle accident injury in patients with obstructive sleep apnea-a nationwide cohort study
Source: BMC Pulm Med. 2020 Feb 3;20:28. doi: 10.1186/s12890-019-1041-1 (PMC6998364; doi:10.1186/s12890-019-1041-1)
Supplement: Supplementary file 7 — Additional file 7: Table S6. Factors of traffic injury by using Cox regression among OSA patients in different treatments. [file 12890_2019_1041_MOESM7_ESM.doc]

| **Table S6. Factors of traffic injury by using Cox regression among OSA patients in different treatments** | |
| --- | --- |
|  | **Adjusted HR (95%CI)** |
| **OSA** | - |
| **Without CPAP or stimulants** | *Reference* |
| **With CPAP only** | 0.81 (95% CI: 0.65-0.96) ^*^ |
| **With stimulants only** | 0.83 (95% CI: 0.67-0.98) ^*^ |
| **With CPAP & stimulants** | 0.69 (95% CI: 0.42-0.88) ^***^ |

**OSA: obstructive sleep apnea; HR: hazard ratio; Adjusted for the variables listed in Table 1**

**^*^ P < 0.05, ^**^ P < 0.01, ^***^ P < 0.001**
